# Frequency-Guided Cross-Modal Interaction for Multimodal Yeast Classification Based on Light-Scattering and Microscopy Images

**DOI:** 10.3390/jimaging12060263

**Published:** 2026-06-16

**Authors:** Zexi Cheng, Xiaoxuan Liu, Shamanth Shankarnarayan, Manisha Gupta, Wojciech Rozmus, Ying Yin Tsui, Daniel A. Charlebois, Mrinal Mandal

**Affiliations:** 1 Department of Electrical and Computer Engineering, University of Alberta, Edmonton, AB T6G 2V4, Canada; zcheng5@ualberta.ca (Z.C.); xliu6@ualberta.ca (X.L.); mgupta1@ualberta.ca (M.G.); ytsui@ualberta.ca (Y.Y.T.); 2 Department of Physics, University of Alberta, Edmonton, AB T6G 2E1, Canada; sashanka@ualberta.ca (S.S.); wrozmus@ualberta.ca (W.R.); dcharleb@ualberta.ca (D.A.C.)

**Keywords:** light scattering imaging, microscopy imaging, frequency-domain analysis, multimodality deep learning, pathogenic yeast, multiclass classification

## Abstract

Accurate identification of pathogenic yeasts is essential for clinical diagnosis and effective antifungal therapy. However, current approaches predominantly rely on microscopy-based models, which require large-scale annotated datasets and exhibit limited generalization across morphologically similar species. In contrast, light-scattering (LS) imaging captures the diffraction patterns generated by internal cellular structures, providing volumetric biophysical cues that extend beyond surface morphology, yet its indirect representations pose major challenges for feature discrimination. Our objective is to develop fast and accurate methods to detect various species of yeasts. We propose FPA-YeastNet, which is a frequency-enhanced single-modality deep learning architecture that improves yeast classification in LS images by leveraging discriminative frequency-domain features. Building upon this enhanced modality, we further propose FGCA-YeastNet, a frequency-guided cross-attention network designed to integrate LS and microscopy information for complementary representation learning. The proposed multimodal model facilitates synergistic interactions between volumetric scattering structures and fine-grained cellular textures through adaptive fusion and bidirectional attention, leading to improved robustness and interpretability. Comprehensive classification experiments conducted on a multimodal yeast dataset demonstrate that FGCA-YeastNet effectively bridges the performance gap between LS and microscopy modalities, achieving significant improvements over both unimodal and multimodal baselines. The FPA-YeastNet yields an average accuracy improvement of 6.26% compared with LS-only models, and FGCA-YeastNet further provides mean gains of 19.97% and 7.67% over unimodal and multimodal baseline models, respectively. Experimental results demonstrate the diagnostic potential of light scattering and microscopic imaging and underscore the effectiveness of frequency-guided multimodal collaboration for reliable and interpretable yeast classification in clinical microbiology.

## 1. Introduction

Pathogenic yeasts are among the most common opportunistic pathogens, responsible for both superficial and invasive infections that pose severe challenges to global healthcare systems [1,2]. These infections can range from mild mucosal lesions to life-threatening bloodstream infections, particularly in immunocompromised patients such as those undergoing chemotherapy, organ transplantation, or immunosuppressive therapy. Therefore, accurate and timely identification of yeast species is crucial in clinical microbiology, as antifungal susceptibility, virulence potential, and therapeutic response vary substantially across species. For instance, *Candida albicans* infections often respond well to azole antifungals, whereas *Candidozyma auris* (previously *Candida auris*) frequently exhibits intrinsic and acquired multidrug resistance, with emerging pan-drug-resistant strains reported in clinical settings [3]. Consequently, developing rapid and accurate classification methods for pathogenic yeasts can directly improve antifungal therapy selection, shorten diagnostic turnaround time, and help mitigate the global spread of drug-resistant strains.

Traditional diagnostic approaches such as culture-based, biochemical assays and PCR-based molecular tests remain the clinical standard. However, these methods are time-consuming, costly, and require specialized facilities and skilled personnel [4,5]. Moreover, they are often ineffective at differentiating morphologically similar yeast species in a clinically relevant timeframe, leading to potential misidentification or delayed treatment [2]. In response, recent studies have explored machine learning and deep learning techniques for automated yeast identification using imaging data. Most of these works have focused on microscopy images, leveraging convolutional neural networks (CNNs) such as ConvNeXt v2 [6], Swin Transformer [7], and MambaVision [8] to classify yeast cells based on morphological features [9,10]. While these methods achieve promising accuracy on large, balanced datasets, they tend to overfit in small-sample microbial datasets and exhibit limited generalization to morphologically similar species.

Light-scattering (LS) imaging has emerged as a promising label-free modality capable of capturing the diffraction and interference patterns generated by subcellular refractive-index variations [11,12]. Recent studies have further demonstrated the growing potential of LS-related imaging and deep learning for label-free cell analysis, including single-cell lymphoma classification, LS-based modal expansion cytometry, and scattering-mitigated high-throughput imaging [13,14,15]. Unlike conventional microscopy, which emphasizes surface morphology and staining-based textures, LS encodes intrinsic biophysical structure through characteristic scattering rings and interference fringes, providing complementary volumetric cues that are invisible to standard optical imaging [16]. Despite these advantages, current deep-learning approaches remain limited in their ability to fully exploit the diagnostic potential of LS images. Most CNN or Transformer-based models treat LS data as conventional intensity images, overlooking their unique spatial-frequency properties. Based on the diffraction principles of LS imaging, we posit that the data exhibits a characteristic frequency distribution: low-frequency components primarily reflect background illumination, high-frequency regions are often dominated by acquisition noise, while mid-frequency bands encode the most informative scattering structures related to cellular morphology. This frequency-dependent characteristic, which has been further empirically validated by our ablation studies, suggests that standard deep learning models may be suboptimal. Nevertheless, informative frequency-band cues may present several challenges when applied to classification architectures. Because frequency representations are globally distributed, they may lose precise spatial correspondence with local morphological structures, limiting spatial localization capability. In addition, scattering distributions can vary under different acquisition conditions, illumination settings, and environmental perturbations, potentially introducing instability into frequency-based representations. Variations in cell size and morphology may also alter the spectral distribution patterns, making it difficult for fixed-frequency priors to generalize consistently across different samples. Furthermore, correlations and interdependencies between frequency components may complicate the separation of informative spectral cues from noise-related responses. These challenges suggest that effectively utilizing frequency-domain information in LS analysis requires robust feature-learning strategies that can balance discriminative spectral cues with robustness to noise and environmental perturbations. Existing attention mechanisms such as SE [17] and CBAM [18] perform feature reweighting purely in the spatial or channel domains, relying on global pooling or convolutional filters that cannot explicitly model frequency-domain characteristics. As a result, these models often underutilize discriminative mid-frequency cues and remain sensitive to illumination bias and high-frequency noise, leading to suboptimal feature discrimination and limited generalization. These challenges highlight the need for specialized frequency-domain representations that can selectively enhanced informative scattering patterns and suppress irrelevant spectral components in LS images.

Meanwhile, with the growing interest in multimodal learning, integrating complementary imaging modalities has shown great promise in biomedical analysis [19]. For example, combining MRI and PET has improved neuroimaging-based disease diagnosis [20], while CT–MRI fusion enhances tumor delineation and characterization [21]. These works highlight the value of combining complementary imaging modalities for more reliable biomedical analysis. However, despite the rapid development of multimodal biomedical learning, most existing studies mainly focus on conventional imaging combinations such as MRI–PET, CT–MRI, or multimodal histopathology analysis. In contrast, the joint modeling of light-scattering (LS) and microscopy modalities remains largely underexplored, particularly for pathogenic yeast classification and frequency-aware multimodal representation learning. Building on this insight, our study jointly models LS and microscopy modalities through a multimodal learning framework to enhance species-level identification of pathogenic yeasts. This combination is particularly valuable, as LS captures intrinsic biophysical and structural cues, whereas microscopy provides phenotypic and textural information. However, achieving robust multimodal fusion in this context remains challenging because the two imaging modalities differ substantially in their underlying physical principles, leading to heterogeneous data distributions that complicate feature alignment and joint representation learning. Moreover, conventional fusion strategies such as simple concatenation or content-driven cross-attention are often insufficient because they do not account for the frequency-dependent structural priors inherent to LS images. At the same time, the limited amount of paired LS–microscopy image data restricts the capacity of deep models to establish stable cross-modal correspondence, especially when morphologically similar yeast species exhibit overlapping structural patterns [10]. Recent studies on robust data processing and noise-aware learning have shown the importance of improving model stability under degraded imaging conditions and noisy supervision. For instance, FNIRNet introduces a lightweight spatiotemporal statistical feature-learning framework for low-channel fNIRS brain function classification, demonstrating the effectiveness of integrating complementary temporal and statistical representations while maintaining computational efficiency [22]. In addition, correntropy-inspired cross-entropy has been proposed to improve robustness against noisy labels [23]. These studies further suggest that robust representation learning is important when dealing with challenging or potentially noisy biomedical imaging data. However, these methods mainly focus on degraded natural images or noisy-label learning, rather than exploiting the frequency-dependent characteristics of LS images or modeling LS–microscopy complementarity.

To address these challenges, we first present FPA-YeastNet, a frequency-enhanced single-modality framework that improves LS-based yeast classification by exploiting informative frequency-band cues. Building on this modality, we then introduce FGCA-YeastNet, a frequency-guided multimodal architecture that integrates LS and microscopy representations through a coarse-to-fine fusion strategy. The model establishes stable global alignment between modalities while progressively refining fine-grained correspondence via frequency-aware modulation, resulting in more complementary and robust representation learning.

The main contributions are summarized as follows:

1. FPA-YeastNet is proposed to enhance yeast classification from LS images. It incorporates a frequency-perception attention (FPA) to emphasize informative structural bands and preserve discriminative mid-frequency features.
2. FGCA-YeastNet is proposed to jointly model LS and microscopy modalities. It introduces a coarse-to-fine attention module (CFAM) comprising a bidirectional cross-attention (BiCAM) for mutual feature guidance and a frequency-guided cross-attention (FGCA) for aligning frequency-domain priors with spatial representations.
3. Experimental results on seven clinically relevant yeast species demonstrate notable improvements over unimodal and multimodal baselines, validating the proposed approach’s generalization ability and clinical diagnostic potential.

## 2. Materials and Methods

This study uses two complementary datasets of pathogenic yeasts: LS images and bright-field microscopy images. The LS dataset contains single-cell angular scattering patterns from seven clinically important species (Table 1). Each image corresponds to a single yeast cell measured using a laser-based side-scattering system with a collection cone angle from 79 to 101 degrees with respect to the laser path under label-free and standardized conditions [12]. The dataset covers a wide morphological range (approximately 1–15 μm) and includes both drug-sensitive and drug-tolerant/resistant isolates [24].

Due to the relatively limited size of the LS dataset and the moderate class imbalance among different yeast species, several strategies were adopted to reduce the risk of overfitting and biased learning. Specifically, random rotation, flipping, and intensity scaling were applied to improve feature diversity and robustness against variations in scattering patterns. In addition, oversampling was applied to underrepresented LS classes during training to alleviate class imbalance and reduce the risk of biased decision boundaries toward majority classes. Furthermore, all LS and microscopy images were resized to a unified spatial resolution (224 × 224), which helped reduce scale variations among different yeast species and improved the consistency of frequency-domain analysis. To avoid misleading performance interpretation caused by dataset imbalance, multiple evaluation metrics, including Accuracy, Precision, Recall, and F1-score, were considered.

**Figure 1:**
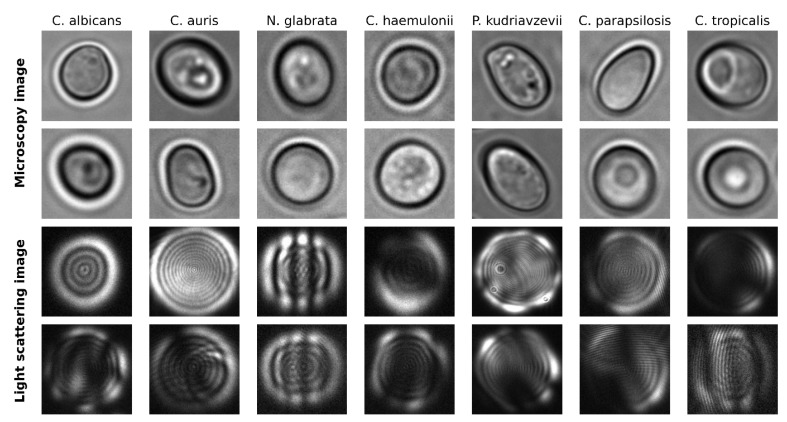
Representative microscope images and light-scattering patterns collected from the 7 species of pathogenic yeast.

Microscopy images were acquired following previously established laboratory procedures [9]. The yeast collection consisted of both clinical isolates and standard reference strains. Clinical isolates of *C. albicans*, *C. auris*, *N. glabrata*, and *C. haemulonii* were provided by Alberta Precision Laboratories—Public Health Laboratory (Edmonton, AB, Canada). In addition, reference strains of *C. parapsilosis* (ATCC 22019), *P. kudriavzevii* (ATCC 6258), and *C. tropicalis* (ATCC 750) were obtained from the American Type Culture Collection (ATCC) (Cedarlane, Burlington, ON, Canada). Prior to imaging, all yeast samples were preserved in 25% glycerol at −80 °C and cultured on Sabouraud Dextrose Agar (SDA) plates (Millipore, Darmstadt, HE, Germany, #1.05438.0500) (Table 1). All microscopy images were also resized to 224 × 224 pixels to ensure consistent input dimensions across modalities. Representative microscopy images and their corresponding LS patterns for the seven yeast species are shown in Figure 1, illustrating the distinct morphological textures captured by microscopy and the complementary angular scattering signatures provided by the LS modality.

## 3. Methodology

### 3.1. Overview

**Figure 2:**
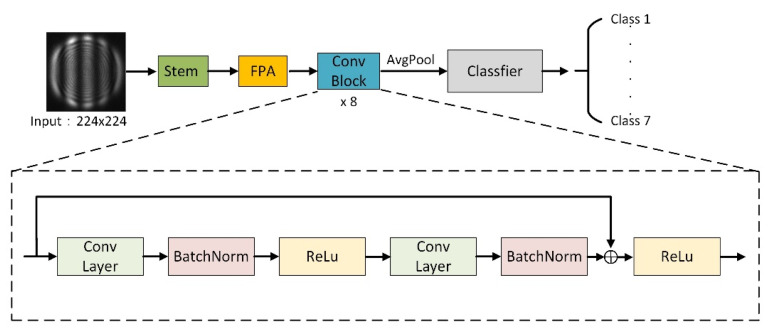
Overview of FPA-YeastNet architecture.

**Figure 3:**
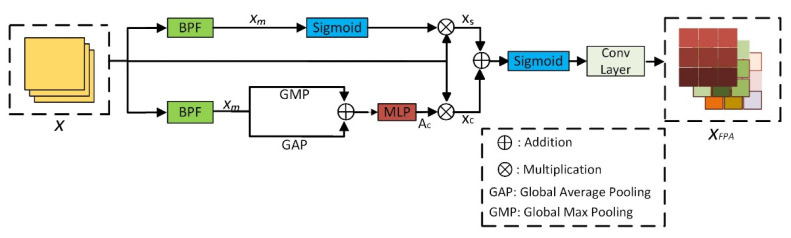
Overview of FPA module architecture.

**Figure 4:**
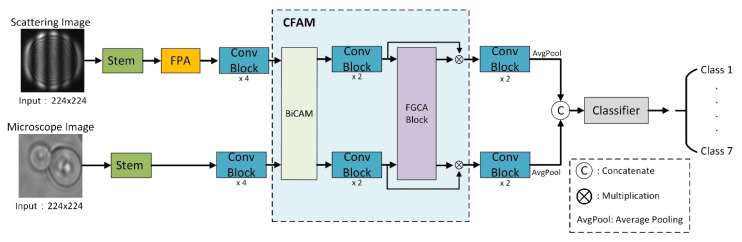
Overall architecture of the proposed FGCA-YeastNet framework.

This work introduces a two-stage framework for yeast classification using LS and microscopy images. The framework consists of two models designed at different representational levels. The first stage is FPA-YeastNet shown in Figure 2, which is a single-modality LS network that incorporates a frequency-domain refinement module (Figure 3) into its feature-extraction backbone. The second stage extends the single modality framework to a multimodal setting through FGCA-YeastNet shown in Figure 4, which jointly processes LS and microscopy data. The CFAM introduced in Figure 4 is composed of two cross-attention submodules to enable gradual interaction and fusion between modalities. These components are described below in detail.

### 3.2. FPA-YeastNet (Single-Modality Baseline)

FPA-YeastNet is a convolutional architecture tailored for LS image analysis. As shown in Figure 2, the network adopts a compact CNN-based architecture consisting of a stem layer, a frequency-perception attention (FPA) module, a sequence of convolutional blocks, and a final classifier. The detailed layer-by-layer configuration, including the input and output tensor shapes at each stage, is summarized in Table 2. The overall workflow of the proposed network is summarized in Algorithm 1.


**Algorithm 1** FPA-YeastNet for Light-Scattering Classification
**Require:** Light-scattering image *I_s_***Ensure:** Predicted yeast category *y*
1:Begin2:Resize input image to 224 *×* 2243:Extract shallow features using Stem layer:4:   *F*_0_ ← Stem(*I_s_*)5:Apply Frequency Pattern Attention (FPA):6:   *F*_1_ ← FPA(*F*_0_)7:Pass features through stacked convolution blocks:8:   *F*_2_ ← ConvBlock(*F*_1_)9:Repeat for 8 convolution blocks10:Perform global average pooling:11: *z* ← AvgPool(*F*_2_)12:Predict yeast category using classifier:13: *y* ← Classifier(*z*)14:Return *y*15:End



#### 3.2.1. Stem Layer

The stem layer serves as the initial feature extractor that transforms the input RGB images into a compact and informative representation. Specifically, it consists of a convolutional block followed by batch normalization, ReLU activation, and max pooling. The convolutional layer uses a 7 × 7 kernel with a stride of 2 and padding of 3, expanding the feature dimension from 3 to 64 while reducing the spatial resolution by half. The subsequent 3 × 3 max pooling layer with stride 2 further downsamples the feature maps to one-quarter of the original input size.

This design, similar to the initial stage of ResNet [7], helps to efficiently capture low-level textures and structural cues from an input image while reducing computational cost for the following stages. The output of the stem layer serves as the input to the FPA module for further frequency-aware processing.

#### 3.2.2. Frequency Perception Attention Module

To enhance scattering-specific patterns in the LS modality, the FPA module is inserted after the stem layer of FPA-YeastNet. As illustrated in Figure 3, the FPA module consists of two parallel branches: a spatial attention branch (top) and a channel attention branch (bottom). Given an input feature map $x\in R^{C\times H\times W}$, the FPA module first performs frequency-domain filtering to selectively preserve informative mid-band components. Specifically, we compute the 2D Fourier transform (FT) of x and apply an annular mask M that retains only the desired frequency range determined by the inner and outer cutoffs. The masked spectrum is then transformed back to the spatial domain using an inverse *FFT* (*IFFT*) to obtain the frequency-enhanced response:

(1) $$x_{m}\left(c\right)=IFFT\left(M⊙FFT\left(x\left(c\right)\right)\right)$$

For the spatial branch, $x_{m}$ is passed through a sigmoid activation to generate a spatial attention map, which is then element-wise multiplied with the original feature map to obtain the spatially modulated representation [29],

(2) $$x_{s}=\;x⊙\sigma (x_{m})$$

where ⊙ represents element-wise multiplication.

For the channel branch, global average pooling (*GAP*) and global max pooling (*GMP*) are applied on the frequency-enhanced feature $x_{m}$ to aggregate global statistics. The two pooled vectors are added and passed through a two-layer MLP followed by a sigmoid activation to generate channel attention weights $A_{c}\in R^{C\times 1\times 1}$:

(3) $$A_{c}=\sigma \left(MLP\left(GAP\left(x_{m}\right)+GMP\left(x_{m}\right)\right)\right)$$

The final channel-refined feature is obtained as

(4) $$x_{c}=A_{c}⊙x$$

with broadcasting along spatial dimensions.

The output of the two branches is fused in the spatial domain and refined by a 3 × 3 convolution:

(5) $$x_{FPA}={Conv}_{3\times 3}\left(\sigma \left(x_{c}+x_{s}\right)\right)$$

The output $x_{\mathrm{FPA}}$ is then forwarded to the subsequent convolutional blocks of FPA-YeastNet, where it serves as a frequency-enhanced feature representation.

#### 3.2.3. Conv Block

The FPA-YeastNet model includes 8 Conv-Blocks (as shown in Figure 2). These Conv-Blocks are designed to progressively extract higher-level semantic representations while maintaining stable gradient propagation across layers.

Each block adopts a residual bottleneck-style structure consisting of two sequential convolutional layers, each followed by batch normalization and ReLU activation. Specifically, the first convolution layer is configured with a stride of 2 when downsampling is required (i.e., for Block 3, 5, 7), thereby reducing the spatial resolution of the feature map by half. The second convolution layer maintains a stride of 1, preserving the spatial resolution while refining and fusing the extracted features to enhance nonlinear representation. A residual connection adds the input feature to the block’s output, enabling efficient feature reuse and mitigating gradient vanishing during training. Table 2 summarizes the input and output shapes for all 8 Conv-Blocks.

#### 3.2.4. Classifier

The output of the last Conv-Block is passed through an average pooling system, resulting in a final feature vector of size 512. This feature vector is then passed through a classifier. The feature vector is first processed by a multi-layer perceptron (MLP) for nonlinear feature refinement, followed by a fully connected layer that projects the refined representation into the class space, producing the final logits *y* (of size 1 × C) corresponding to the yeast species. These logits y are normalized by a softmax function to produce the predicted class probabilities, defined as

(6) $$p_{i}=\frac{\exp\left(y_{i}\right)}{\sum_{j=1}^{C}exp(y_{j})},i=1,\ldots ,C,$$

where $p_{i}$ denotes the predicted probability for class i, and C  represents the total number of yeast species.

To improve model calibration and enhance the stability of predictions among morphologically similar classes, we employ the cross-entropy (CE) loss with label smoothing (ϵ=0.05). The loss function is formulated as

(7) $$L_{CE,LS}=-\frac{1}{N}\sum_{i=1}^{N}\sum_{c=1}^{C}{[(1-\epsilon )y}_{i,c}+\frac{\epsilon }{C}\;]\log\left({\tilde{y}}_{i,c}\right)$$

where N is the number of training samples, ${\tilde{y}}_{\left(i,c\right)}$ is the predicted probability that sample i belongs to class c, and $y_{\left(i,c\right)}\in \{0,1\}$ is the one-hot ground-truth indicator. The smoothing factor ϵ softens the target distribution by allocating a small portion of probability mass to all non-target classes, which mitigates over-confident predictions, improves calibration, and yields more consistent decision boundaries between visually similar yeast species.

### 3.3. FGCA-YeastNet (Extended Multimodal Framework)

The FGCA-YeastNet extends FPA-YeastNet by incorporating microscopy images into a multimodal architecture. As illustrated in Figure 4, the network builds upon the LS branch of FPA-YeastNet, reusing its feature-extraction backbone and classifier described in Section 3.2. The overall architecture consists of two feature extraction branches for LS and microscopic images, a CFAM, and a classifier. The overall workflow of the proposed FGCA-YeastNet is summarized in Algorithm 2.
**Algorithm 2** FGCA-YeastNet for Multimodal Yeast Classification**Require:** Microscopy image *I_m_*, light-scattering image *I_s_***Ensure:** Predicted yeast category *y*1:Begin2:Extract modality-specific features:3:   *F_s_* ← FPA(Stem(*I_s_*))4:   *F_m_* ← Stem(*I_m_*)7:Pass features through stacked convolution blocks:8:   *F_s_*, *F_m_* ← ConvBlock(*F_s_, F_m_*)6:Perform bidirectional coarse alignment using BiCAM:7:   *F*ˆ*m*, *F*ˆ*s* ← BiCAM(*F_m_*, *F_s_*)8:Apply frequency-guided cross-attention:12: *F*˜*m*, *F*˜*s* ← FGCA(*F*ˆ*m*, *F*ˆ*s*)12:Refine features using convolution blocks:13: *G_s_* ← ConvBlock(*F*˜*s*)14: *G_m_* ← ConvBlock(*F*˜*m*)15:Perform global average pooling:16: *P_s_* ← AvgPool(*G_s_*)17: *P_m_* ← AvgPool(*G_m_*)18:Concatenate pooled features:19: *F_fusion_* ← Concat(*P_s_*, *P_m_*)12:Predict yeast category using classifier:13: *y* ← Classifier(*F_fusion_*)14:Return *y*15:End

#### 3.3.1. Coarse-to-Fine Attention Module (CFAM)

The CFAM integrates the LS and microscopy branches through two sequential stages. The first stage applies a BiCAM layer, enabling reciprocal feature querying to establish an initial semantic alignment between modalities. The second stage employs an FGCA block, which refines the fused representation by modulating attention weights using the frequency-energy characteristics of LS features. This hierarchical design enables stable coarse alignment followed by frequency-aware refinement, yielding more discriminative and consistent multimodal representations.

##### Bi-Directional Cross-Attention Module

As the first stage of the proposed CFAM, the BiCAM performs coarse-level semantic alignment between the two modalities before frequency-guided refinement.

**Figure 5:**
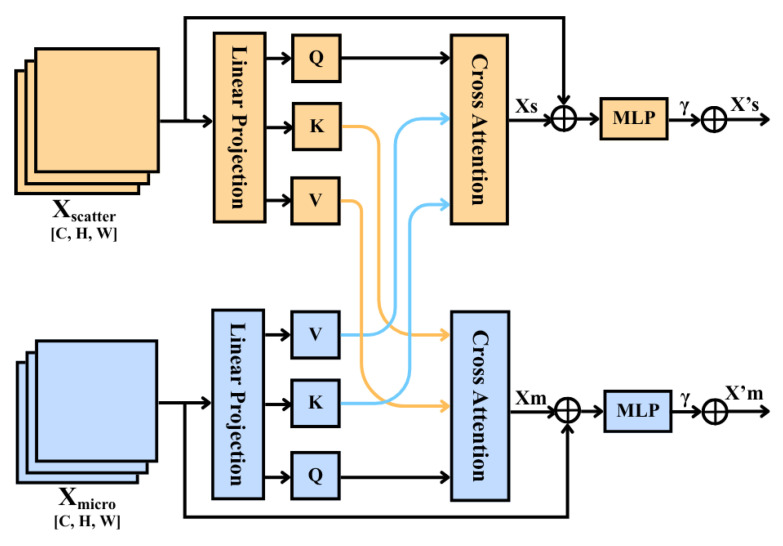
Overall architecture of the proposed BiCAM. The module performs bidirectional cross-modal interaction between light-scattering and microscopy features through two symmetric cross-attention branches. In the upper branch, scatter features provide the query (Q), while microscopy features provide the key (K) and value (V), enabling scatter-guided attention. The outputs are further refined through residual connections and MLP layers to enhance multimodal feature fusion.

As illustrated in Figure 5, the proposed BiCAM is inspired by the transformer architecture [30] and adopts a bidirectional cross-attention design to enable reciprocal interaction between light-scattering and microscopic features. Specifically, the upper branch performs micro-to-scatter attention, where microscopy features guide the refinement of scattering representations, while the lower branch performs scatter-to-micro attention, where scattering features are used to update microscopy representations. Unlike conventional single-direction fusion, the proposed design allows the two modalities to exchange complementary information simultaneously through two parallel cross-attention paths.

**(a) Micro-to-Scatter Attention (** ${CA}_{m\to s}$ **)**

In the upper branch, microscopic features provide fine-grained cues to refine the scattering representation. Given the modality-specific feature maps $X_{\mathrm{scatter}},X_{\mathrm{micro}}\in R^{C\times H\times W}$, they are first flattened into token sequences and then linearly projected to obtain the query, key, and value embeddings:

(8) $$Q_{\mathrm{scatter}}=X_{\mathrm{scatter}}W^{q},K_{\mathrm{micro}}=X_{\mathrm{micro}}W^{k},V_{\mathrm{micro}}=X_{\mathrm{micro}}W^{v}$$

where $W^{q},W^{k},W^{v}\in R^{C\times d_{k}}$ are learnable projection matrices and the cross-attention is computed as

(9) $$X_{s}={\mathrm{CA}}_{m\to s}(Q_{\mathrm{scatter}},K_{\mathrm{micro}},V_{\mathrm{micro}})=\mathrm{softmax}\;(\frac{Q_{\mathrm{scatter}}K_{\mathrm{micro}}^{T}}{\sqrt{d_{k}}})V_{\mathrm{micro}}$$

where the scaling factor $d_{k}$ stabilizes the dot-product magnitude during training. This enables microscopic features to selectively attend to scattering features based on contextual similarity.

**(b) Scatter-to-Micro Attention (** ${CA}_{s\to m}$ **)**

Conversely, in the lower branch, scattering features provide fine-grained cues to refine the microscope representation. The process is symmetric, where the query is derived from the microscopy tokens, the key and value are obtained from the scattering tokens:

(10) $$Q_{\mathrm{micro}}=X_{\mathrm{micro}}W^{q},K_{\mathrm{scatter}}=X_{\mathrm{scatter}}W^{k},V_{\mathrm{scatter}}=X_{\mathrm{scatter}}W^{v}$$

and the cross-attention is computed as:

(11) $$X_{m}={\mathrm{CA}}_{s\to m}(Q_{\mathrm{micro}},K_{\mathrm{scatter}},V_{\mathrm{scatter}})=\mathrm{softmax}\;(\frac{Q_{\mathrm{micro}}K_{\mathrm{scatter}}^{T}}{\sqrt{d_{k}}})V_{\mathrm{scatter}}$$

**(c) Residual and Gated Fusion**

Finally, the attention outputs are fused with their original query features through residual connections and further refined by a Gated MLP:

(12) $$X_{s,m}^{\prime }=X_{s,\;m}+\gamma \cdot MLP(X_{s,\;m})$$

where γ is a learnable gating scalar controlling the contribution of the FFN branch. The token sequence $X_{s,m}^{\prime }$ is then reshaped back to the 2D feature format $R^{C\times H\times W}$.

##### Frequency-Guided Cross-Attention Block

**Figure 6:**
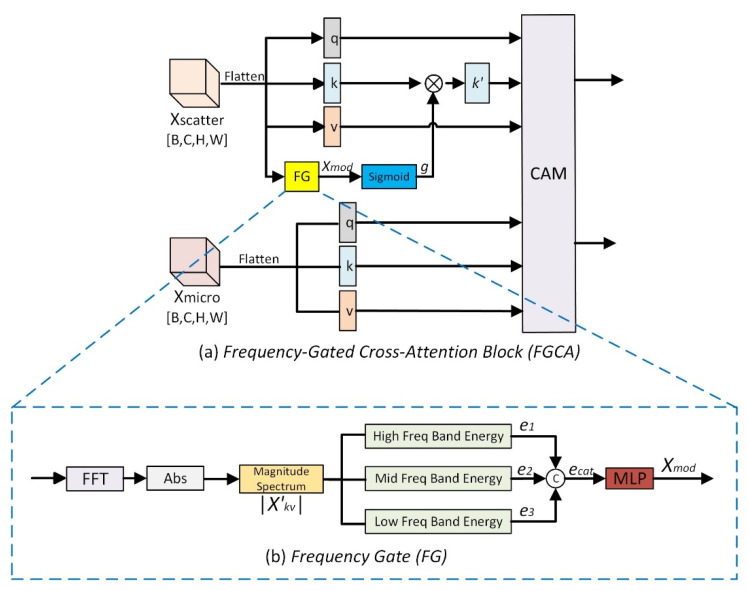
Overall architecture of the proposed FGCA module. (**a**) the Frequency-Gated Cross-Attention block; (**b**) the Frequency Gate Module used for modulation.

Following the initial semantic alignment established by BiCAM, the FGCA block performs frequency-guided refinement to enhance cross-modal fusion. FGCA integrates a Frequency Gate (FG) module that modulates the keys of the cross-attention operation (Figure 6a). Specifically, scattering features are first transformed into a frequency-energy descriptor through the FG module (Figure 6b), which produces a gating vector used to reweight the key representations. The reweighted keys are then fed into the CAM, enabling the attention weights to preferentially focus on frequency-informative scattering components during multimodal fusion. This mechanism enables FGCA to incorporate domain-specific spectral priors from LS data into the attention process, yielding more discriminative and stable multimodal representations.

Formally, given a scattering feature map $X_{scatter}\in R^{C\times H\times W}$, we first compute its Fourier magnitude spectrum channel-wisely:

(13) $$X_{kv}^{\prime }=|FFT\left(X_{scatter}\right)|$$

and partition it into three frequency bands using concentric annular masks ${\left\{M_{k}\right\}}_{k=1}^{3}$, which correspond to the low, mid, and high frequency regions respectively. For the kth band, the per-channel average energy is

(14) $$e_{k}=\frac{{\sum \left(M_{k}⊙X_{kv}^{\prime }\right)}_{h,w}}{\sum {\left(M_{k}\right)}_{h,w}+\epsilon },e_{k}\in R^{C}$$

The concatenated energy vector

(15) $$e_{cat}=[e_{1},\ldots ,e_{k}]$$

is mapped through a lightweight MLP to yield the modulation vector:

(16) $$X_{mod}=MLP(e_{cat}),{\;X}_{mod}\in R^{C}$$

As shown in Figure 6a, a gating signal is then generated as

(17) $$g=\sigma (X_{mod})$$

where *σ* denotes the sigmoid function. With a microscopy query feature $x_{q}$, and key/value projections *k*, *v* derived from the flattened scattering feature map, the gated keys are defined as

(18) k′=k⊙g

where broadcasting is applied along the sequence dimension.

The frequency gate is applied only to the keys, as modulating the key embeddings effectively guides the attention distribution while preserving the semantics of both modalities. The subsequent attention computation follows the standard formulation described in Section 3.3.1, with the original keys k replaced by their gated counterparts $k^{\prime }$. By selectively enhancing scattering features according to their spectral energy distribution, the FGCA block introduces a frequency-aware inductive bias into multimodal fusion.

## 4. Experiments

To evaluate the effectiveness and generalization capability of the proposed framework, we conducted comprehensive experiments on the multimodal yeast classification task involving both LS and microscopic images. We first describe the experimental settings used in our study. Then, we present the quantitative results of the proposed method and compare its performance with several state-of-the-art approaches. In addition, ablation experiments are conducted to verify the contribution of each key component.

### 4.1. Experimental Setup

#### 4.1.1. Evaluation Metrics

In this study, we report Accuracy (Acc), Precision, Recall, and F1-score to comprehensively evaluate model performance in a multi-class setting. For a problem with K classes, the per-class precision, recall, and F1-score are computed using a one-vs-all strategy for per-class metric computation, where each class k is treated as the positive class and all remaining classes as negative.

Formally, for each class *k*

(19) $${\mathrm{Precision}}_{k}=\frac{TP_{k}}{TP_{k}+FP_{k}}$$

(20) $${\mathrm{Recall}}_{k}=\frac{TP_{k}}{TP_{k}+FN_{k}}$$

(21) $${F1}_{k}=\frac{2\times {\mathrm{Precision}}_{k}\times {\mathrm{Recall}}_{k}}{{\mathrm{Precision}}_{k}+{\mathrm{Recall}}_{k}}$$

Overall accuracy is defined as

(22) $$\mathrm{Accuracy}=\frac{\sum_{k=1}^{K}TP_{k}}{\sum_{k=1}^{K}\left(TP_{k}+FP_{k}+FN_{k}\right)}$$

Here, $TP_{k}$, $FP_{k}$, and $FN_{k}$ denote the number of true positives, false positives, and false negatives of class k, respectively. In all experiments, Precision, Recall, and F1-score are reported in their macro-averaged form, where metrics are computed independently for each class and then averaged with equal weight.

We also evaluate the computational efficiency of the proposed model in terms of the number of trainable parameters and floating-point operations (GFLOPs). These two indicators reflect the model’s complexity and inference cost, providing a fair comparison of both accuracy and computational burden across different architectures.

#### 4.1.2. Training Configuration

We adopted a five-fold cross-validation strategy to ensure a fair and reliable evaluation. The entire dataset was first partitioned into five mutually exclusive and approximately equal-sized folds while preserving class balance. In each fold, three folds were used for training, one-fold for validation, and the remaining fold for testing. This process was repeated five times so that every sample served as a test instance exactly once. The final performance was reported as the mean across all folds.

All experiments were conducted using PyTorch 2.5.1 and Python 3.12 with CUDA 12.4. The model was trained on a single NVIDIA RTX 3090 GPU (24 GB) equipped with an Intel Xeon Gold 6330 CPU. Training was performed for 100 epochs with a batch size of 16. The AdamW optimizer [31] was used with an initial learning rate of 1 × 10^−4^ and a weight decay of 1 × 10^−3^. The cross-entropy loss with label smoothing (ϵ = 0.05) [32] was employed as the objective function.

### 4.2. Quantitative Evaluation

#### 4.2.1. Performance Comparison of FPA-YeastNet (Unimodal Baseline)

To ensure fairness, all comparison methods were configured identically except for their network architectures. We evaluated three categories of single-modal models: convolutional neural networks (CNNs), transformer-based architectures, and state-space sequence models. Among CNNs, DenseNet201 [33] achieved the best performance on LS images. For transformers, ViT [34] and Swin Transformer [7] were employed to capture global and hierarchical local representations. The MambaVision [35] model was further tested to assess the potential of sequential modeling for visual dependencies, offering a complementary perspective beyond convolutional and attention mechanisms.

Table 3 summarizes the performance of the proposed FPA-YeastNet and representative unimodal baselines trained solely on LS images. All unimodal models, including CNN, Transformer, and Mamba-based architectures, achieve F1-scores between 60% and 67%, suggesting their limited ability to model the structural information embedded in scattering patterns. In contrast, our proposed FPA-YeastNet attains an average accuracy of 70.00% and F1-score of 69.55%, outperforming the strongest baseline (ViT) by 3.71% in F1.

Overall, the results verify that FPA-YeastNet substantially enhances the discriminative capacity of label-free LS imaging, serving as a solid foundation for the proposed multimodal framework.

#### 4.2.2. Performance Comparison of FGCA-YeastNet (Multimodal Framework)

Building upon the frequency-enhanced LS representation achieved by FPA-YeastNet, the proposed FGCA-YeastNet extends the framework into a multimodal setting by incorporating microscopy images through frequency-guided cross-attention. All multimodal and microscopy-only models were trained under identical conditions, differing only in their architectural configurations.

We evaluated representative single-modal microscopy models and state-of-the-art multimodal fusion architectures for comparison. Among unimodal microscopy models, DenseNet201 [33] achieved the highest F1-score of 87.67%, followed by MambaVision [35] and Swin Transformer [7]. These results indicate that, while microscopy images provide richer morphological information, performance improvements plateau even with more advanced architectures. Notably, the fact that transformer-based models did not surpass DenseNet may be attributed to their heavy reliance on large-scale training data, which limits the generalization capability of transformer-based models in this specific task.

**Figure 7:**
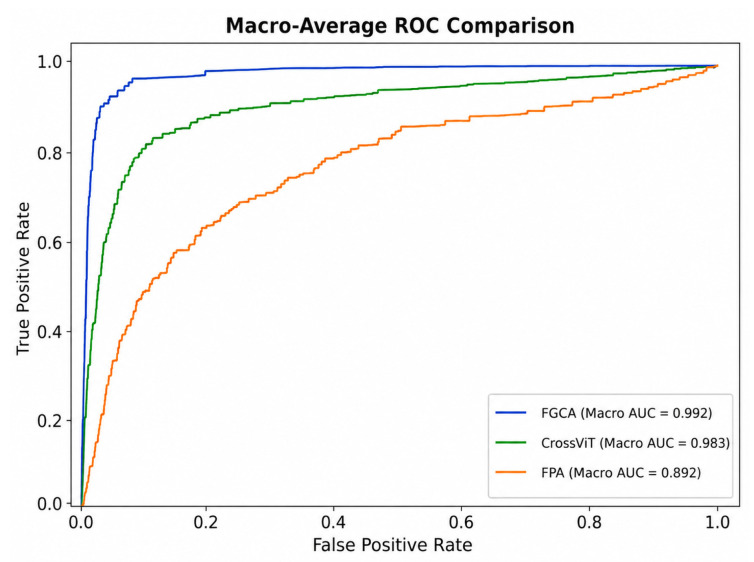
Macro-average ROC comparison among FGCA-YeastNet, CrossViT, and FPA-YeastNet on the yeast classification dataset.

As shown in Table 4, FGCA-YeastNet achieves the best overall performance with an accuracy of 94.78% and F1-score of 94.76%, surpassing all competing methods. Compared to the strongest microscopy-based model DenseNet201, FGCA-YeastNet yields absolute gains of 6.82% in accuracy and 7.09% in F1-score. Furthermore, compared to the leading multimodal baselines CrossViT [36] and SwinFuse [37], the proposed model achieves improvements of 4.91% and 10.43% in accuracy, and 6.48% and 12.57% in F1-score, respectively. The relatively lower performance of SwinFuse [37] may be attributed to domain discrepancies; SwinFuse was designed for infrared and visible image fusion therefore its feature interaction mechanisms may not be optimal for the specific characteristics of our dataset. Figure 7 further illustrates the macro-average ROC comparison among the proposed multimodal FGCA-YeastNet, the single-modal FPA-YeastNet, and CrossViT, which represents the second-best state-of-the-art baseline in terms of classification performance. As shown in the figure, FGCA-YeastNet achieves the highest AUC and exhibits the steepest ROC curve near the upper-left corner, indicating superior discriminative capability and classification consistency.

#### 4.2.3. Computational Efficiency Analysis

Table 5 compares the computational efficiency of the proposed models with representative unimodal and multimodal baselines. Overall, FPA-YeastNet is among the most lightweight models, requiring substantially fewer parameters and GFLOPs than conventional CNN, Transformer, and state-space architectures, demonstrating that frequency-domain enhancement can be achieved with minimal computational overhead.

For the multimodal setting, FGCA-YeastNet achieves a favorable balance between efficiency and performance. Despite incorporating dual branches and cross-modal attention, its parameter count and computational cost remain noticeably lower than transformer-based multimodal models, while delivering the highest accuracy and F1-score among all competitors. These results confirm that the proposed frequency-guided fusion mechanism offers significant performance gains without sacrificing computational scalability.

### 4.3. Ablation Study

#### 4.3.1. Ablation Study for Single Modality FPA-YeastNet

To verify the effectiveness of the frequency-domain prior introduced by the FPA module, we conducted an ablation study analyzing the impact of different frequency band ranges on model performance.

**Figure 8:**
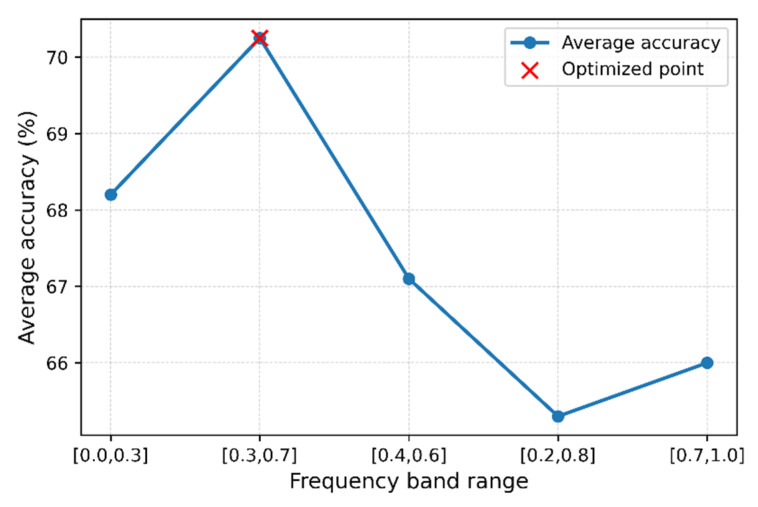
Average classification accuracy under different frequency band ranges used in the Frequency-Perception Attenuation module.

Specifically, we varied the inner and outer cutoff ratios $\left(r_{\mathrm{inner}},r_{\mathrm{outer}}\right)$ used to define the annular frequency masks in the Fourier magnitude spectrum. The tested configurations include five representative ranges. Figure 8 summarizes the average classification accuracy achieved under each setting.

The proposed model attains the highest average accuracy when the mid-frequency range [0.3, 0.7] is selected (Figure 8). Interestingly, we observed that performance degrades when the bandwidth is either tightened to [0.4, 0.6] or expanded to [0.2, 0.8]. This suggests that an overly narrow bandwidth may discard valuable structural cues, whereas an excessively broad range likely introduces noise and redundancy. In contrast, low-frequency bands ([0.0, 0.3]) mainly encode coarse intensity variations, while high-frequency bands ([0.7, 1.0]) are more sensitive to noise and microscopic artefacts, both leading to inferior performance. The ablation trend demonstrates a clear mid-frequency dominance, validating the design choice of FPA to emphasize mid-band spectral responses as the primary information carrier for scattering-based morphological representation. In addition, the frequency-domain information is not directly used for standalone classification but rather employed as an auxiliary feature-enhancement cue within the attention framework. Consequently, the model still preserves substantial spatial contextual information from the original feature maps, reducing sensitivity to slight frequency shifts caused by variations in yeast size or imaging conditions.

Together, the ablation study quantitatively confirms that the FPA module’s mid-band focus is not arbitrary but empirically optimal, reinforcing its role as an effective frequency-domain prior for feature enhancement in LS analysis.

#### 4.3.2. Ablation Study for Multimodal FGCA-YeastNet

We also conducted ablation experiments to analyze the independent contribution of each key module in FGCA-YeastNet (Figure 4). These components include the FPA, BiCAM, and FGCA.

Four model variants were evaluated, and their results are shown in Table 6. FPA alone noticeably improves the baseline, confirming its ability to enhance frequency-aware LS representations. Adding BiCAM on top of FPA brings further gains by strengthening bidirectional cross-modal interaction. The combination of BiCAM and FGCA achieves even higher performance than FPA and BiCAM, as FGCA directly refines cross-modal fusion through frequency-guided attention. Finally, integrating all three components yields the best accuracy and F1-score, indicating that FPA, BiCAM, and FGCA provide complementary rather than redundant contributions.

In addition, to evaluate the influence of input modality, we compared single and dual modality settings in Table 7. It can be observed that using both LS and microscopy modalities achieves the best overall performance. While microscopy alone achieves higher accuracy, LS contributes complementary structural cues that are absent in microscopy, and their combination yields the best results. Specifically, our multimodal strategy outperforms the single-modality counterparts by at least 7.87% in accuracy and 8.07% in F1-score, demonstrating the crucial role of cross-modal complementarity in enhancing classification robustness and generalization.

### 4.4. Statistical Analysis

To validate the robustness of the observed improvements, we conducted paired *t*-tests for both the unimodal and multimodal settings. Specifically, FPA-YeastNet was compared against the strongest LS baseline (ViT), while FGCA-YeastNet was evaluated against the best multimodal SOTA model (CrossViT). As shown in Table 8, all resulting *p*-values are below the significance threshold of α = 0.001, indicating that the gains provided by both FPA-based enhancement and the full multimodal fusion are statistically significant. These results confirm that our proposed framework consistently and reliably outperforms its respective state-of-the-art baselines.

### 4.5. Feature Visualization

**Figure 9:**
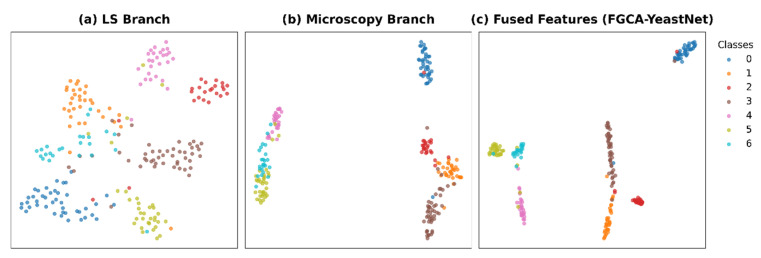
Comparison of t-SNE visualizations of (**a**) LS features, (**b**) microscope features, and (**c**) fused features from FGCA-YeastNet, showing progressively improved cluster separation after cross-modal fusion. The analysis covers seven yeast species indexed as follows: Class 0 (*C. albicans*), Class 1 (*C. auris*), Class 2 (*N. glabrata*), Class 3 (*C. haemulonii*), Class 4 (*P. kudriavzevii*), Class 5 (*C. parapsilosis*), and Class 6 (*C. tropicalis*).

To better understand the complementary properties of LS and microscopy modalities and to evaluate the effectiveness of cross-modal fusion in FGCA-YeastNet, we visualize the high-dimensional features using t-SNE. Figure 9 presents the feature distributions of the LS branch, the microscopy branch, and the fused representation obtained after the FGCA module.

The LS branch exhibits high intra-class variance with dispersed clusters, reflecting the inherently indirect nature of scattering signals (Figure 9a). However, despite local overlaps, it maintains global distinguishability, positioning different cell types into distinct regions of the feature space. In contrast, the microscopy branch (Figure 9b) forms significantly tighter and better-separated clusters, benefiting from its rich spatial resolution. Yet, certain species still exhibit proximity, suggesting that visual morphological similarities remain challenging to differentiate using image data alone. Crucially, the fused features (Figure 9c) demonstrate the clearest inter-class boundaries and the tightest intra-class compactness. This confirms our hypothesis of complementarity: the LS branch provides unique volumetric and refractive signatures—offering a global physical context—while the microscopy branch refines local boundaries with fine-grained texture information. Our proposed FGCA-YeastNet effectively aligns these heterogeneous cues, producing a feature space with superior discriminability.

These observations confirm the intrinsic cross-modal complementarity between LS and microscopy data and further validate that FGCA-YeastNet leverages this complementarity to construct a more robust and semantically coherent representation.

## 5. Discussion

In this work, we validated the complementary roles of microscopy and light-scattering (LS) imaging in pathogenic yeast classification. Our experiments confirm that microscopy primarily captures localized 2D morphological cues such as surface texture, shape, and cellular boundaries, resulting in high intra-class compactness. In contrast, LS encodes angle-dependent scattering responses influenced by internal refractive-index variations. While LS signals exhibit higher intra-class variance due to their indirect nature, they provide critical global distinguishability through distinctive interference-fringe patterns (e.g., horizontal, vertical, and concentric structures). These optical signatures, which are absent in standard microscopy, effectively prevent class overlap among morphologically similar species.

The proposed FPA-YeastNet and FGCA-YeastNet leverage these complementary properties through a frequency-aware fusion strategy. FPA-YeastNet enhances LS representation by emphasizing mid-frequency components, which we identified as striking the optimal balance between high-frequency noise and low-frequency redundancy. Building on this, FGCA-YeastNet integrates the modalities via a coarse-to-fine attention mechanism. BiCAM establishes a stable global alignment, while FGCA further refines cross-modal correspondence using frequency priors from LS images. This design effectively mitigates the heterogeneity between spatial morphological features (microscopy) and frequency-derived scattering patterns (LS). As evidenced by the t-SNE visualization, the fused embedding combines the global separability of LS with the local compactness of microscopy, producing a semantically coherent feature space with significantly enhanced discriminability.

## 6. Conclusions

In this study, we proposed a frequency-enhanced multimodal framework for pathogenic yeast classification. By introducing FPA-YeastNet, we improved LS-based recognition through the selective emphasis of mid-band spectral features. Furthermore, FGCA-YeastNet extends this capability to a multimodal setting via frequency-guided cross-attention, effectively aligning heterogeneous optical cues. Extensive experiments on seven clinically relevant yeast species demonstrate that our framework consistently outperforms both unimodal and state-of-the-art multimodal baselines. These results highlight the diagnostic potential of combining morphological imaging with label-free scattering signatures, offering a rapid and robust solution for microbial identification. Ultimately, this study paves the way for advanced multimodal representation learning in biological analysis, particularly for tasks requiring fine-grained differentiation of structurally similar cells.

## Figures and Tables

**Table 1:** Distribution of light-scattering and microscopic image dataset for pathogenic yeast species. Approximate cell size ranges for each yeast species were obtained from established clinical microbiology literature, including reports on *Candida albicans*, *Nakaseomyces glabrata*, *Candida parapsilosis* and *Candida tropicalis* [25], *Candidozyma auris* [26], *Candida haemulonii* [27], and *Pichia kudriavzevii* [28].

No.	Species	No. of Light Scattering Images	No. of Microscopic Images	Cell Size Range (μm)
1	*Candida albicans*	278	400	4.0–10.0
2	*Candidozyma auris*	218	400	2.0–3.0
3	*Nakaseomyces glabrata*	156	400	1.0–4.0
4	*Candida haemul* *onii*	314	400	2.0–3.0
5	*Pichia kudriavzevii*	182	400	2.2–15.2
6	*Candida parapsilosis*	222	400	2.5–9.0
7	*Candida tropicalis*	162	400	4.0–11.0
Total		1532	2800	

**Table 2:** Layer-by-layer architecture of the proposed FPA-YeastNet.

Module	Input Shape	Output Shape
Stem	[3, 224, 224]	[64, 56, 56]
FPA	[64, 56, 56]	[64, 56, 56]
ConvBlock1&2	[64, 56, 56]	[64, 56, 56]
ConvBlock3	[64, 56, 56]	[128, 28, 28]
ConvBlock4	[128, 28, 28]	[128, 28, 28]
ConvBlock5	[128, 28, 28]	[256, 14, 14]
ConvBlock6	[256, 14, 14]	[256, 14, 14]
ConvBlock7	[256, 14, 14]	[512, 7, 7]
ConvBlock8	[512, 7, 7]	[512, 7, 7]
AvgPool	[512, 7, 7]	[512, 1, 1]
Classification Head	[512, 1, 1]	[7]

**Table 3:** Performance comparison (mean ± std) of the proposed FPA-YeastNet with State-of-the-Art (SOTA) models on LS images. The best results are highlighted in bold.

Model	Acc	Pre	Rec	F1
DenseNet201 [27]	64.56 ± 0.07	65.26 ± 0.07	65.74 ± 0.07	64.61 ± 0.07
Resnet-50 [7]	62.65 ± 0.04	63.75 ± 0.04	64.72 ± 0.04	62.53 ± 0.05
ViT [28]	66.38 ± 0.05	66.88 ± 0.05	65.42 ± 0.05	65.84 ± 0.04
Swin Transformer [29]	64.19 ± 0.04	64.99 ± 0.04	66.48 ± 0.04	63.72 ± 0.03
MambaVision [30]	60.91 ± 0.13	61.61 ± 0.17	62.29 ± 0.15	60.52 ± 0.16
**Ours (FPA-YeastNet)**	**70.00 ± 0.12**	**70.25 ± 0.08**	**69.72 ± 0.09**	**69.55 ± 0.10**

**Table 4:** Performance comparison (mean ± std) of the proposed model with single and multi-modal SOTA models. The best results are highlighted in bold.

Modality	Model	Acc	Pre	Rec	F1
Microscope	DenseNet201 [27]	87.96 ± 0.07	88.08 ± 0.06	87.86 ± 0.07	87.67 ± 0.07
Microscope	Resnet-50 [7]	83.71 ± 0.04	84.81 ± 0.03	84.52 ± 0.03	83.76 ± 0.04
Microscope	ViT [28]	84.86 ± 0.04	85.19 ± 0.04	85.00 ± 0.04	84.10 ± 0.04
Microscope	Swin Transformer [29]	85.24 ± 0.04	86.00 ± 0.03	85.95 ± 0.03	85.11 ± 0.04
Microscope	MambaVision [30]	87.65 ± 0.11	87.77 ± 0.15	87.14 ± 0.13	87.25 ± 0.14
LS + Microscope	CrossViT [31]	89.87 ± 0.03	89.18 ± 0.04	88.4 ± 0.03	88.28 ± 0.04
LS + Microscope	SwinFuse [32]	84.35 ± 0.04	82.81 ± 0.04	83.35 ± 0.04	82.19 ± 0.06
LS + Microscope	**Ours (FGCA-YeastNet)**	**94.78 ± 0.08**	**94.85 ± 0.10**	**94.90 ± 0.09**	**94.76 ± 0.11**

**Table 5:** Efficiency comparison in terms of parameters and Giga Floating Point Operations (GFLOPs) per second.

Model	Parameters (M)	GFLOPs
DenseNet201 [27]	20.0	4.29
Resnet-50 [7]	23.5	4.09
ViT [28]	86.6	17.56
Swin Transformer [29]	87.8	15.43
MambaVision [30]	50.1	7.50
CrossViT [31]	43.3	9.00
SwinFuse [32]	10.2	26.08
Ours (FPA-YeastNet)	11.2	1.94
Ours (FGCA-YeastNet)	26.2	5.29

**Table 6:** Ablation studies for the key components of our FGCA-YeastNet. The best results are highlighted in bold.

FPA	BiCAM	FGCA	Acc	F1
			86.70	85.99
✓			89.21	88.59
✓	✓		91.43	91.22
	✓	✓	92.74	91.32
✓	✓	✓	**94.78**	**94.76**

**Table 7:** Performance comparison between single-modality and dual-modality input in FGCA-YeastNet. The best results are highlighted in bold.

LS Image	Microscope Image	Acc	F1
✓		70.00	69.55
	✓	84.91	84.69
✓	✓	**94.78**	**94.76**

**Table 8:** Significance test results between the proposed model and the best SOTA baseline.

Model	Compared Baseline	Metrics	*p*-Value	Significance
FPA-YeastNet	ViT	Acc	<0.001	✓
		Pre	<0.001	✓
		Rec	<0.001	✓
		F1	<0.001	✓
FGCA-YeastNet	CrossViT	Acc	<0.001	✓
		Pre	<0.001	✓
		Rec	<0.001	✓
		F1	<0.001	✓

## Data Availability

The data presented in this study are available upon reasonable, non-commercial request from the corresponding author.
